# PERCEPTIONS OF COMMUNICATION: INCLUSION OF DEMENTIA CARE PARTNERS IN HOSPITALIZED PATIENT CARE

**DOI:** 10.1093/geroni/igae098.0446

**Published:** 2024-12-31

**Authors:** Austin Medlin, Catherine Still, Andrea Strayer, Beth Fields, Nicole Werner

**Affiliations:** Indiana University Bloomington, Bloomington, Indiana, United States; University of Wisconsin Madison, Madison, Wisconsin, United States; University of Iowa, Iowa City, Iowa, United States; University of Wisconsin Madison, Madison, Wisconsin, United States; Indiana University, Bloomington, Bloomington, Indiana, United States

## Abstract

Care partners provide the majority of care to people living with dementia (PLWD) during and after hospitalizations. Yet, they often report dissatisfaction with their inclusion in the hospitalization and feel unprepared to provide care after hospital discharge. Effective communication between clinicians and care partners of PLWD during a hospitalization is foundational to care partner inclusion and preparation. However, our current understanding of care partner communication with hospital-based clinicians is lacking. The present study aims to explore care partners’ experiences with clinician communication during hospitalizations of PLWD. A qualitative descriptive study was conducted using semi-structured interviews with 15 care partners of PLWD who had experienced a hospitalization within the last year. Our interdisciplinary team completed an inductive content analysis of the interview transcripts focused on identifying factors influencing care partner and clinician communication. Excerpts were first structurally coded related to hospital communication and then overarching categories were generated through consensus-based discussion over multiple team meetings. We identified five distinct categories related to care partner communication with clinicians: amount of communication, care transition communication, communication style, care partner communication expectations, and inclusion of PLWD in communication. Negative perceptions arose surrounding physical presence of the care partner dictating amount of communication, while lack of communication between hospitals and home health led to decreased care transition communication. Those with positive experiences, however, described a “family-like” communication style. These findings point to areas of communication that could be targets for future intervention to improve care partner inclusion and preparation during hospitalizations of PLWD.

